# Pregnancy in Sickle Cell Disease Is a Very High-Risk Situation: An Observational Study

**DOI:** 10.1155/2016/9069054

**Published:** 2016-06-15

**Authors:** Narcisse Elenga, Aurélie Adeline, John Balcaen, Tania Vaz, Mélanie Calvez, Anne Terraz, Laetitia Accrombessi, Gabriel Carles

**Affiliations:** ^1^Pediatric Unit, Centre Hospitalier de Cayenne “Andrée Rosemon”, rue des Flamboyants, BP 6006, 97306 Cayenne Cedex, French Guiana; ^2^Integrated Center of Sickle Cell Disease (ICS), Centre Hospitalier de Cayenne “Andrée Rosemon”, rue des Flamboyants, BP 6006, 97306 Cayenne Cedex, French Guiana; ^3^Department of Medical Information, Centre Hospitalier de Cayenne “Andrée Rosemon”, rue des Flamboyants, BP 6006, 97306 Cayenne Cedex, French Guiana; ^4^Obstetrics and Gynecology Unit, Centre Hospitalier de Cayenne “Andrée Rosemon”, rue des Flamboyants, BP 6006, 97306 Cayenne Cedex, French Guiana; ^5^Obstetrics and Gynecology Unit, Centre Hospitalier de l'Ouest Guyanais Franck Joly, 16 boulevard du Général de Gaulle, BP 245, 97393 Saint-Laurent-du-Maroni, French Guiana

## Abstract

Sickle cell disease is a serious genetic disorder affecting 1/235 births in French Guiana. This study aimed to describe the follow-up of pregnancies among sickle cell disease patients in Cayenne Hospital, in order to highlight the most reported complications. 62 records of pregnancies were analyzed among 44 females with sickle cell disease, between 2007 and 2013. Our results were compared to those of studies conducted in Brazil and Guadeloupe. There were 61 monofetal pregnancies and 2 twin pregnancies, 27 pregnancies among women with SS phenotype, 30 SC pregnancies, and five S-beta pregnancies. The study showed that the follow-up of patients was variable, but no maternal death was found. We also noted that the main maternofetal complications of pregnancies were anemia (36.5%), infection (31.7%), vasoocclusive crisis (20.6%), preeclampsia (17.5%), premature birth (11.1%), intrauterine growth retardation (15.9%), abnormal fetal heart rate (14.3%), and intrauterine fetal death (4.8%). Pregnancies were more at risk among women with SS phenotype. Pregnancy in sickle cell disease patients requires a supported multidisciplinary team including the primary care physician, the obstetrician, and the Integrated Center for Sickle Cell Disease.

## 1. Introduction

Sickle cell disease is the most common inherited disorder worldwide with varying clinical severity and potentially serious complications [1]. Pregnancy in sickle cell disease is at very high risk. Many reports have documented a considerable maternal risk of morbidity and mortality and high perinatal adverse outcomes [2–7]. Recently, Oteng-Ntim et al. [8], in a systematic review and meta-analysis of previous observational studies, have quantified this risk. They showed that women with SCD have an increased risk of preeclampsia and maternal death, stillbirths, preterm deliveries, and small-for-gestational-age newborns. Knowledge of these risks has contributed to the implementation of a multidisciplinary management program including the early detection and treatment of complications during pregnancy and postpartum, follow-up by an obstetric team and a sickle cell team, appropriate pain management protocols, and transfusion programs adapted to each pregnant patient.

In French Guiana, a French overseas territory in South America, SCD has an incidence of 1/235 births. There are 6,000 deliveries each year and the number of pregnancies among women with sickle cell disease is estimated at 20 per year. The two main public hospitals in French Guiana each have a gynecology and obstetrics unit with obstetricians experienced in the management of sickle cell disease. Monitoring of patients with sickle cell disease occurs in the outpatient unit by sickle cell disease specialists. Management of SCD during pregnancy is multidisciplinary and involves aggressive interventions such as transfusion or exchange transfusion in patients with high-risk pregnancy.

## 2. Materials and Methods

### 2.1. Study Type

This was a retrospective and descriptive study that aimed to analyze the evolution of pregnancies in patients with sickle cell disease during the period from January 1, 2007, to December 31, 2013.

### 2.2. Study Participants

The subjects were patients with sickle cell pregnancies followed up at the Cayenne Hospital in French Guiana. The inclusion criterion was a pregnancy in a SCD patient (HbSS, HbS-beta, or HbSC diagnosed by hemoglobin electrophoresis) occurring between 2007 and 2013. The patients were first divided into three groups according to their genotypes, in order to evaluate differences in sickle cell complications during pregnancy by genotype. We also evaluated the impact of blood transfusion on sickle cell complications during pregnancy. The transfusion program was adapted to each pregnant patient, and women received blood transfusion only if the hemoglobin fell below 8 g/dL and in case of obstetrical or sickling complications. Prophylactic transfusion regimens were available for sickle cell patients with a history of serious obstetrical and/or sickling complications. This protocol consisted of a transfusion every 15 days or exchange transfusion every 21 days, from 24 to 26 weeks of gestation for up to 36 weeks.

### 2.3. Design

Clinical data were obtained through a review of medical records from the hospital with the confidentiality of information being preserved. Laboratory test results were obtained through the online hospital system using our hospital software. These data were recorded in an Excel spreadsheet and later compiled for statistical analysis.

This data collection was approved by the Commission Nationale Informatique et Libertés (CNIL) number 1896086 v 0, which is a national committee that oversees research data.

### 2.4. Data Analysis

Data are presented as descriptive statistics with means and percentages. The Mann-Whitney nonparametric statistical test was used to compare the transfused/nontransfused groups. Data analysis was performed using Epi Info*™* 7.

## 3. Results

There were 62 pregnancies occurring in 44 women: three women delivered 3 times (14.5%), 12 women delivered 2 times (38.7%), and 29 others were primiparous (46.8%). The mean age was 26.4 years (17–41).  Table 1 shows the characteristics of the patients.

Among obstetrical complications (Table 2), there were a 41% prematurity percentage in the SS genotype against 22% for SC, 11% of pregnant women with miscarriages in SS against 3% for SC, and 3 in utero fetal deaths in homozygous women. No maternal death was noted. In Cayenne Hospital, there were 17,729 deliveries between 2007 and 2013. Six maternal deaths in the perinatal period were identified. The causes of these deaths were preeclampsia (2 cases), puerperal sepsis (2 cases), disseminated intravascular coagulation (1 case), and puerperal cardiomyopathy (1 case).

Nonobstetric complications are shown in Table 3. There was a similar frequency of acute chest syndrome and urinary tract infection in SS and SC parturients.

Neonatal complications (Table 4) led to hospitalization in neonatal intensive care unit in 15% of SS patients against 7% for SC patients. Intrauterine growth retardation and abnormal fetal heart rate were frequent.

Comparison between patients who have received prophylactic transfusion compared to curative transfusion showed no difference in terms of maternofetal morbidity and mortality (Table 5).

Comparison of our results with other studies (Table 6) shows that, despite progress in the treatment of sickle cell disease, pregnancy remains a situation with very high risk for both the mother and the fetus.

## 4. Discussion

Pregnancy in a sickle cell woman is at very high risk, especially in patients with more severe sickle cell disease (HbSS genotype, a history of cerebral vasculopathy, and acute chest syndrome (ACS)). Indeed, the risk of maternal and fetal complications is higher than in the general population [8]. Chronic fetal hypoxia associated with decreased placental circulatory flow level seems the most plausible explanation for this high incidence of perinatal complications [11].

This study, like many already published observational ones, confirms this high complication rate. Indeed, there is in our study a high rate of maternal (cesarean section, preeclampsia, premature delivery, miscarriage, hospitalization in intensive care unit, severe VOC, and ACS) and fetal (in utero fetal death, premature birth, and hospitalization in neonatal intensive care unit) complications. We found no maternal or neonatal death. This bias could be related to the retrospective nature and the low power of the study. Indeed, although they are in constant decline, we still observe from time to time in our hospitals in French Guiana cases of maternal and neonatal deaths in women with sickle cell disease.

This study certainly confirms data already known, but it concerns a recent cohort (2007–2013) from a region of France located in South America, particularly hit by this SCD. Apart from meta-analyses [8], which also most often relate only to retrospective data, most published studies are descriptive, with a limited number of included patients [9–13]. So a prospective study with sufficient power will better characterize these maternal and fetal complications.

Indeed, thanks to significant progress in the management of sickle cell disease, most women now survive to an age when they are able to become pregnant [14, 15]. Thus the number of women with sickle cell disease will continue to grow. Also, pooling data from several cohorts through a prospective multicenter study could help to confirm and explain the risk factors of that maternal and fetal mortality.

We found no difference in prognosis between women who had prophylactic transfusions and those not systematically transfused. Although blood transfusion is a therapeutic method that can sometimes be necessary in severe forms of SCD, the routine prophylactic transfusion regimen does not confer a clear clinical benefit compared to the curative transfusion. Currently, there is no evidence from randomized trials to provide formal guidance on transfusion strategy during pregnancy of women with sickle cell disease [16]. In France, at the present time and in the absence of formal scientific proof, there is a consensus on the importance of selective or systematic prophylactic transfusion from the 22nd week of gestation, every 3 weeks until delivery [17, 18]. Some teams (Henri Mondor University Hospital, e.g.) even advocate beginning of the systematic transfusion at the end of the first quarter in patients with a history of symptomatic sickle cell syndrome, late miscarriage, intrauterine growth retardation (IUGR), or twin pregnancy.

In the literature, there is currently some controversy regarding whether or not to use prophylactic blood transfusion during pregnancy [19–21]. On the pathophysiological level, the intake of normal red blood cells improves maternal and fetal exchanges. Indeed, transfusion brings normal hemoglobin in the bloodstream, thus reducing the circulating sickle cell hemoglobin. The effectiveness of transfusion is also related to the fact that sickle erythrocytes have a shorter lifespan than normal erythrocytes.

Indirectly, the transfusion reduces abnormal erythropoiesis by lowering the medullary stimulus and therefore the production of hemoglobin S by the bone marrow. Finally, the hemoglobin S decrease reduces vascular alterations in the placenta and improves oxygenation of the fetus [11].

In some cases, the systematic oxygen therapy may have a beneficial role in reducing the transfusion indications. Here, again a large-scale prospective study would confirm, with a significant power, the benefit of this oxygen therapy.

It seems that the risk of complications during pregnancy in women with SC genotypes is lower than in the homozygous women but greater than in healthy women [13]. Our results confirm these data. However, we have found a higher frequency of obstetric complications in SC women. This could be explained by the less aggressive treatment offered to these SC parturients.

Given the high risk of maternofetal morbidity, SC parturients would require care and monitoring similar to those of SS [22].

In all cases, a multidisciplinary approach should be implemented from the desire for pregnancy. Then monitoring will be carried out both by an obstetrician trained to treat women with sickle cell disease and by a sickle cell disease specialist [23–25].

## 5. Conclusion

This study confirms that pregnancy in sickle cell women has numerous obstetrical, nonobstetrical, and fetal complications. The results of our study did not show any difference in maternal or fetal morbidity by genotype. Moreover, we have found no difference between women who had prophylactic transfusions and those not systematically transfused. That could be an evidence of effectiveness as one might expect more complications in the first group. Indeed, it compares two different populations, but the criteria for inclusion in the risk group to be transfused remain unclear; these criteria vary according to the teams. Our daily experience shows that preventive transfusion benefits only the most severe forms. A comparison should be performed between 2 groups of patients with the same genotype and the same severity index in order to highlight the transfusion benefit.

Multidisciplinary support in specialized centers should be performed. The benefit of certain therapeutic strategies during pregnancy, such as blood transfusions or oxygen therapy, deserves to be better clarified by multicenter prospective studies.

## Figures and Tables

**Table 1:** Characteristics of the deliveries according to the patients' sickle cell genotype.

	HbSS	HbS/beta	HbSC
Number of deliveries	27	5	30
Mean age (years)	28.2 (17–41)	26.4 (21–31)	24.5 (17–40)
Previous HU use, *n* (%)	8 (30)	0	1 (3)
Blood transfusion, *n* (%)	19 (70)	0	0
Sickle cell complications, *n* (%)	16 (59)	0	10 (33)
Obstetric complications, *n* (%)	11 (41)	4 (80)	17 (57)

**Table 2:** Maternal obstetric complications during pregnancy.

Obstetric complications	HbSS	HbS/beta	HbSC
Previous miscarriage *n* (%)	6 (22)	0	7 (23)
Preterm labor *n* (%)	11 (41)	0	7 (23)
Preeclampsia *n* (%)	3 (11)	2 (40)	6 (20)
Gestational diabetes *n* (%)	0	0	0
Cesarean section *n* (%)	10 (37)	2 (40)	21 (70)
Stillbirth *n* (%)	1 (3.7)	0	3 (10)
Fetal death in utero *n* (%)	3 (11)	0	0
Abortion *n* (%)	3 (11)	0	1 (3)

**Table 3:** Nonobstetric maternal complications during pregnancy.

Nonobstetric complications *n* (%)	HbSS	HbS/beta	HbSC
Severe vasoocclusive crises	5 (19)	0	8 (27)
Acute chest syndrome	1 (4)	0	1 (3)
Urinary tract infection	8 (30)	0	10 (33)
Impaired cardiac function	0	0	0
Hospitalization in intensive care unit			
Maternal death	0	0	0

**Table 4:** Analysis of the characteristics of newborn infants.

Characteristics	HbSS	HbS/beta	HbSC
Mean gestational age (weeks)	34.84 (10–41.6)	38.52 (37.7–39.8)	36.76 (27.1–41)
Average weight (g)	2632 (500–3530)	3214 (2710–3690)	2590 (670–3670)
Five-minute Apgar	8.5 (3–10)	8 (3–10)	9.12 (3–10)
Intrauterine growth retardation *n* (%)	5 (19)	0	10 (33)
Abnormal fetal heart rate *n* (%)	1 (4)	1 (20)	7 (23)
Hospitalization in ICU^*∗*^ *n* (%)	4 (15)	0	2 (7)

^*∗*^ICU: intensive care unit.

**Table 5:** Comparison of maternal-fetal characteristics between transfused and nontransfused pregnant women with sickle cell disease during pregnancy.

	Nontransfused (*n* = 33)	Transfused (*n* = 29)	*p*
Median age (years)	25.9 (16–40)	26 (17–41)	0.7
Pregestational Hb (g/dL)	9.9 (8.4–11.4)	8.7 (7.1–10.7)	0.06
Abnormal fetal heart rate *n* (%)	6 (18)	3 (10)	0.4
Preterm labor *n* (%)	4 (12)	3 (10)	0.3
Preeclampsia *n* (%)	5 (15)	6 (21)	0.8
Fetal death in utero *n* (%)	1 (3)	2 (7)	0.5
Intrauterine growth retardation *n* (%)	8 (24.2)	7 (24.1)	0.9
Maternal hospitalization in ICU *n* (%)	5 (15)	5 (17)	0.8
Maternal death *n* (%)	0	0	
Total death (maternal and fetal) *n* (%)	0	0	
Prematurity *n* (%)	6 (18)	12 (41)	0.04

**Table 6:** Comparison of maternal-fetal characteristics between two published studies and the current study.

	Current study	Silva-Pinto et al. [9]	Leborgne-Samuel et al. [10]
Number of patients	62	34	68
*Obstetric complications (%)*			
Previous miscarriage	21	26.6	
Prematurity	29		21
Intrauterine growth retardation	24.2		8.8
Preeclampsia	17.7	12	10
Perinatal death	5	7.7	6
Cesarean section	53	41	48
Gestational diabetes	0	6	
Neonatal death	0	0	2.9
Maternal death	0	2.9	1.7
